# Correction: A highly selective fluorescent probe for human NAD(P)H:quinone oxidoreductase 1 (hNQO1) detection and imaging in living tumor cells

**DOI:** 10.1039/c9ra90078k

**Published:** 2019-10-25

**Authors:** Ya Zhu, Jialing Han, Qian Zhang, Zhou Zhao, Jin Wang, Xiaowei Xu, Haiping Hao, Jun Zhang

**Affiliations:** School of Pharmacy, China Pharmaceutical University Nanjing 210009 China xw@cpu.edu.cn; School of Pharmacy, Nanjing Medical University Nanjing 211166 China zhangjun@njmu.edu.cn

## Abstract

Correction for ‘A highly selective fluorescent probe for human NAD(P)H:quinone oxidoreductase 1 (hNQO1) detection and imaging in living tumor cells’ by Ya Zhu *et al.*, *RSC Adv.*, 2019, **9**, 26729–26733.

The authors regret that some articles reporting probes for detecting human NAD(P)H:quinone oxidoreductase 1 were not cited in the original article. The missing references are listed below as ref. 1–6, and should be cited in the original paper at the end of the following sentence on page 26729:

Herein, we designed and synthesized a novel fluorescent probe 1 for detection of hNQO1 based on TCF-OH as a chromophore and quinone propionic acid (QPA) as a recognition group.^1–6^

The authors sincerely apologise for this oversight.

The Royal Society of Chemistry apologises for these errors and any consequent inconvenience to authors and readers.

## Supplementary Material
